# Ribbon like appearance of the midsubstance fibres of the anterior cruciate ligament close to its femoral insertion site: a cadaveric study including 111 knees

**DOI:** 10.1007/s00167-014-3146-7

**Published:** 2014-06-28

**Authors:** Robert Śmigielski, Urszula Zdanowicz, Michał Drwięga, Bogdan Ciszek, Beata Ciszkowska-Łysoń, Rainer Siebold

**Affiliations:** 1 Orthopaedic and Sports Traumatology Department, Carolina Medical Center, Pory 78, 02-757 Warsaw, Poland; 2 Department of Descriptive and Clinical Anatomy, Medical University of Warsaw, Chalbinskiego 5, 02-004 Warsaw, Poland; 3 Department of Radiology, Carolina Medical Center, Pory 78, 02-757 Warsaw, Poland; 4 Institute for Anatomy and Cell Biology, INF, Ruprecht-Karls University Heidelberg, Heidelberg, Germany; 5 HKF: Center for Spezialised Hip-Knee-Foot Surgery, ATOS Hospital Heidelberg, Bismarckstr. 9-15, 69115 Heidelberg, Germany

**Keywords:** Ribbon, Anterior cruciate ligament, ACL, Femoral insertion, Intraligamentous, Midsubstance, Anatomy

## Abstract

**Purpose:**

Recently, the configuration of the anterior cruciate ligament (ACL) from its direct femoral insertion to midsubstance was found to be flat. This might have an important impact for anatomical ACL reconstruction. The purpose of this anatomical study was to evaluate the macroscopic appearance of the ACL from femoral to midsubstance.

**Methods:**

The ACL was dissected in 111 human fresh frozen cadaver knees from its femoral insertion to midsubstance, and the shape was described. The anatomical findings were documented on digital photographs and on video. Thirty knees were sent for computed tomography (CT), magnetic resonance imaging (MRI) and histology of the femoral ACL insertion.

**Results:**

Two millimetres from its direct femoral insertion, the ACL fibres formed a flat ribbon in all dissected knees without a clear separation between AM and PL bundles. The ribbon was in exact continuity of the posterior femoral cortex. The width of the ribbon was between 11.43 and 16.18 mm and the thickness of the ACL was only 2.54–3.38 mm. 3D CT, MRI and the histological examination confirmed above findings.

**Conclusion:**

This is a detailed anatomical study describing the ribbon-like structure of the ACL from its femoral insertion to midsubstance. A key point was to carefully remove the surface fibrous membrane of the ACL. A total of 2–3 mm from its bony femoral insertion, the ACL formed a flat ribbon without a clear separation between AM and PL bundles. The ribbon was in exact continuity of the posterior femoral cortex. The findings of a flat ligament may change the future approach to femoral ACL footprint and midsubstance ACL reconstruction and to graft selection.

**Electronic supplementary material:**

The online version of this article (doi:10.1007/s00167-014-3146-7) contains supplementary material, which is available to authorized users.

## Introduction

A deep understanding of the morphology of the anterior cruciate ligament (ACL) is fundamental for its anatomical reconstruction, and most surgeons would agree that anatomical ACL reconstruction is the “restoration of the ACL to its native dimensions, collagen orientation and insertion sites” [18].

From previous anatomical studies, it is well known that the bony femoral ACL insertion is in the shape of a crescent, with the residents ridge (= lateral intercondylar ridge) as its straight anterior border and the posterior articular margin of the lateral femoral condyle as its convex posterior border [3, 5, 6, 9, 10, 13, 15, 19, 21, 23, 36, 39, 41, 43, 45, 52]. Most ACL fibres are aligned posterior to—and directly along the lateral intercondylar ridge. The longitudinal axis is in extension to the posterior femoral cortex and creates an angle to the femoral shaft axis which varies between 0° and 70° [6, 14, 25, 41, 42, 43, 45]. The most posterior fibres of the femoral insertion are blending with the posterior cartilage of the lateral femoral condyle and with the periosteum of the posterior femoral shaft [14, 19, 25, 42, 43, 45]. The femoral insertion site area shows big variations in size. According to the literature, the area varies between 46 and 230 mm^2^, the length between 12 and 20 mm and the width between 5 and 13 mm [6, 10, 14, 19, 21, 24, 25, 29, 36, 42, 45]. Girgis (1975) [19] described the midsubstance of the ACL to be broad and flat with an average width of 11.1 mm. Other authors reported the diameter in the range between 7 and 13 mm and the cross-sectional area to be “irregular”, “oval”, “corded” or “bundled” [2, 4, 6, 13, 19, 27–29, 36, 38, 51].

Recently, detailed observations of the femoral insertion site were reported by Mochizuki et al. [30], Iwahashi et al. [25] and Sasaki et al. [42]. Histologically, they described the ACL midsubstance fibres to form a narrow “direct” insertion posterior and along to the lateral intercondylar ridge which was continued by a fan-like “indirect” insertion towards the posterior femoral cartilage. Interestingly, they described the configuration of the ACL midsubstance to be “rather flat, looking like lasagna” [31].

The detailed understanding on the femoral insertion and the midsubstance shape of the ACL is very important for anatomical ACL reconstruction and may have a significant impact on clinical results.

The purpose of this anatomical cadaveric study was to evaluate the morphology of the ACL from its direct femoral insertion to midsubstance.

## Materials and methods

One hundred and eleven fresh frozen cadaveric knees from 81 humans were used in this anatomical study: 45 male (of which 17 with both knees) and 36 female (of which 13 with both knees) from the MedCure tissue bank in Portland, Oregon, USA). Eleven knees with severe osteoarthritic changes (Grade IV according to the Outerbridge classification of osteochondral injuries [17]) were excluded from the study. Detailed demographic data are presented in Table 1.

**Table 1:** Detailed demographic data of the study subjects

Sex	Side	Age	Height	BMI	Weight	Races
36 Female	49 Right	Mean 67 y (32–74 y)	Mean 1.70 m (1.50–1.96 m)	Mean 22.6 (12.1–34.7)	Mean 64.3 kg (36–116 kg)	104 Caucasians 6 African Americans 1 Indian American
45 Male	62 Left					

Thighs and legs were sectioned approximately 30 cm away from the joint line. Knees were first thawed. All dissections were performed by the first author. All soft tissue superficial structures (anterior, posterior, medial and lateral side) to the level of the joint capsule were removed. The quadriceps tendon and patella with patellar tendon were excised distally at the level of the tibial tuberosity and also removed. After exposing the anterior aspect of the knee joint, the synovial tissue and Hoffa fat pad were carefully dissected and separated from the articular soft tissue structures (menisci and transverse ligament). Using an oscillating saw, the medial femoral condyle was cut through the intercondylar notch and was removed for better visualisation of the ACL and its femoral attachment. The key point in the dissections was the very careful and accurate removal of the synovial tissue surrounding the collagen fibres of the ACL.

After achieving good visualisation of the knee joint and ACL, anthropometric measurements of the ACL were taken 2 mm from its bony insertion and at midsubstance. Digital photographs and video recording were performed by a professional photographer using a Canon EOS 1 with a 24–70 mm lens. Measurements were made of the knee with the vertical tibia supported by the table and the femur manually fixed at full extension, as this is when the ACL achieves its maximum length (1). Measurements were performed under direct visualisation using vernier calipers (VIS, Poland).

Thirty knees were then sent for CT and MRI scans as well as histological examination of the femoral insertion site. CT scans were performed in eight different positions of the knee: from full extension and 20°, 40°, 60°, 90°, 110°, 130° and 150° of flexion and were made at 120 kV, 300 mAs, with 0.67 mm slice thickness; pitch 0.66 (Brilliance CT 40-channel). The knees were covered with plastic, put on their side on the CT table and adjusted to different degrees of flexion with a goniometer. Confirmation of exact knee position (flexion) was made on the basis of an overview (pilot) CT scan. The CT data—volume rendering (VR) and multi-planar reconstruction (MPR)—were processed with Philips Brilliance CT applications (Philips, Netherlands).

Magnetic resonance imaging (MRI) examinations were performed in full extension (MR: 1.5, TSigna HDxt, GE Medical Systems machine, USA) using an 8 channel HD Knee Array in the following sequences: axial PD FSE TR/TE 2600-2800/24–30 ms; matrix 512 × 320; slice thickness 1.1 mm Gap 0 mm; Nex 5; sagittal PD FSE TR/TE 2800/24–30 ms; matrix 512 × 320; slice thickness 2 mm Gap 0 mm; Nex 3; axial PD multi-planar reconstruction and processed with the Carestream Client.

The CT scans and the MRIs were performed to evaluate and reconfirm the macroscopic appearance of the midsubstance fibres of the ACL close to its femoral insertion. Histologies were performed using a light microscopy, H&E stain and a 4 × -magnification to investigate the femoral ACL insertion including the proximal midsubstance fibres of the ACL close to its femoral insertion site.

The study was performed in the Department of Descriptive and Clinical Anatomy, in the Center for Biostructure Research, Medical University of Warsaw.

## Results

**Fig. 1:**
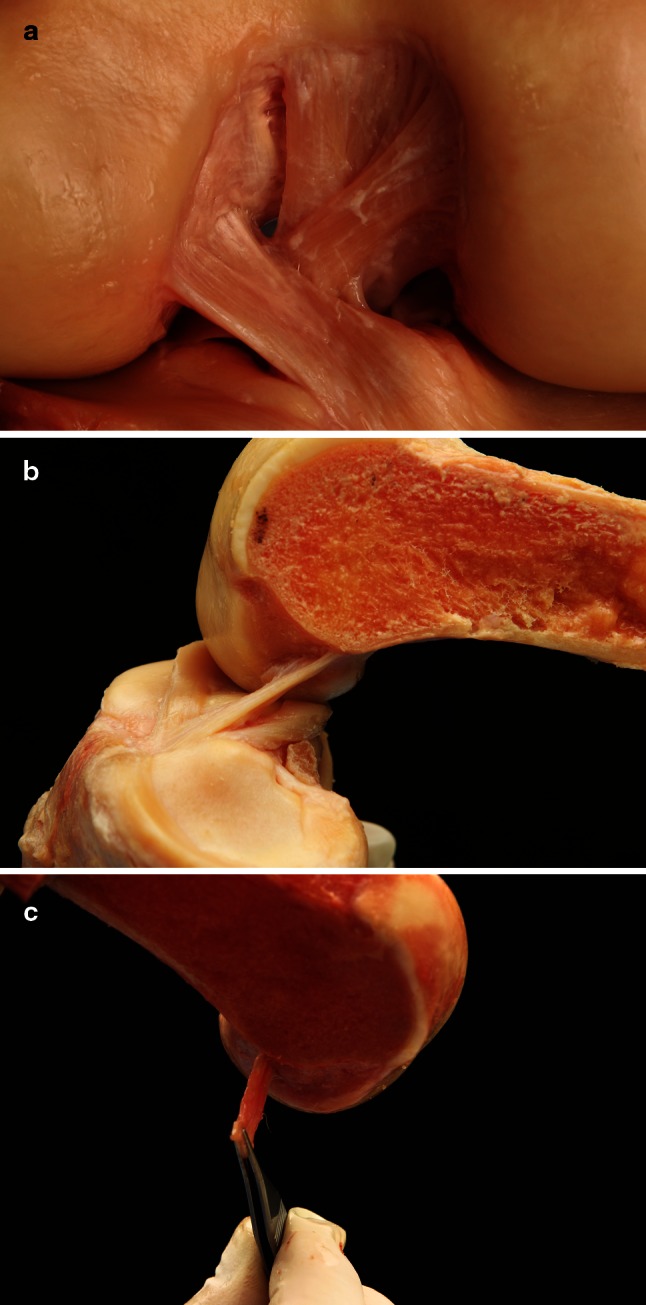
**a**–**c** The ribbon shape of the ACL after careful removal of the synovial tissue: The ACL fibres form a flat ribbon 2 mm from its femoral attachment to midsubstance

**Fig. 2:**
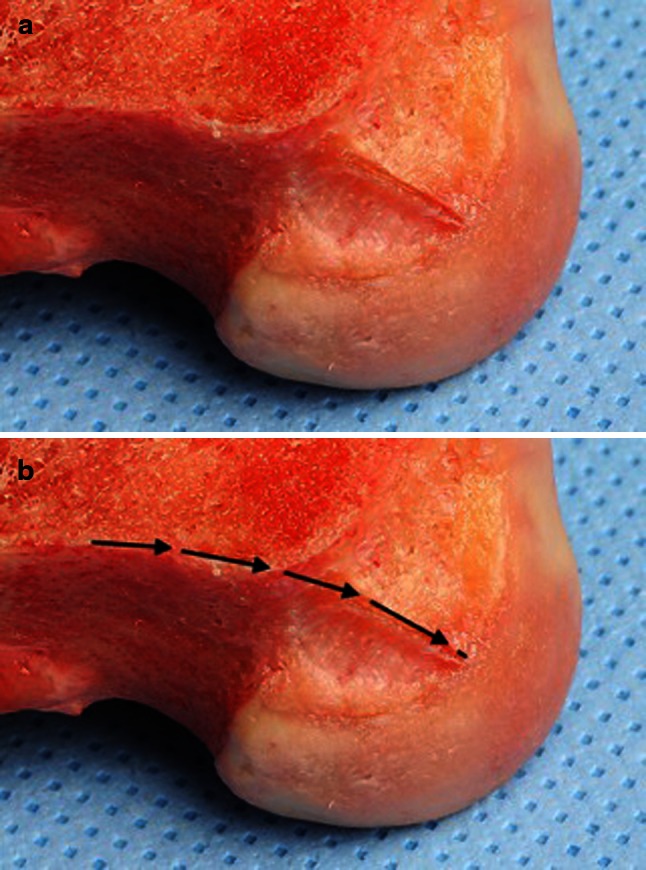
**a**, **b** The direct insertion of the ribbon-like ACL fibres is in continuity of the posterior femoral cortex

**Fig. 3:**
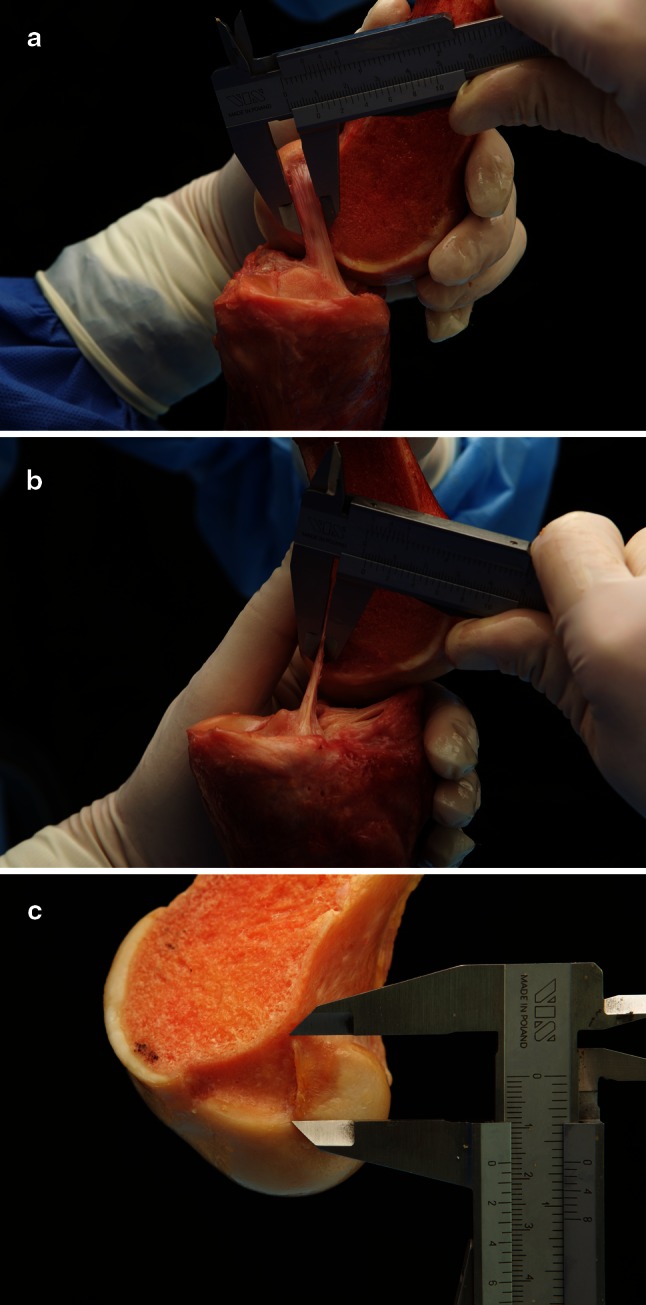
**a**–**c** Measurement of the midsubstance width, thickness and long axis of the ACL using a calliper

In all dissected knees, the intraligamentous part of the ACL from close to its femoral insertion to midsubstance was observed to have a ribbon-like structure (Fig. 1a–c, Video 1). The femoral bony insertion of the ribbon was in exact continuity to the posterior femoral cortex (Fig. 2a, b). A clear separation into bundles was not possible. The morphometric measurements of the ACL were performed with calipers. The results for the width and thickness were as follow (Fig. 3a–c):

- Mean width 2 mm from femoral insertion: 16.0 mm (range 12.7–18.1)
- Mean thickness 2 mm from femoral insertion: 3.54 mm (range 2–4.8)
- Mean cross-sectional area 2 mm from femoral insertion (calculated): 56.6 mm^2^
- Mean width at midsubstance of ACL: 11.4 mm (range 9.8–13.8).
- Mean thickness at midsubstance of ACL: 3.4 mm (range 1.8–3.9).
- Mean cross-sectional area at midsubstance of ACL (calculated): 39.8 mm^2^

**Fig. 4:**
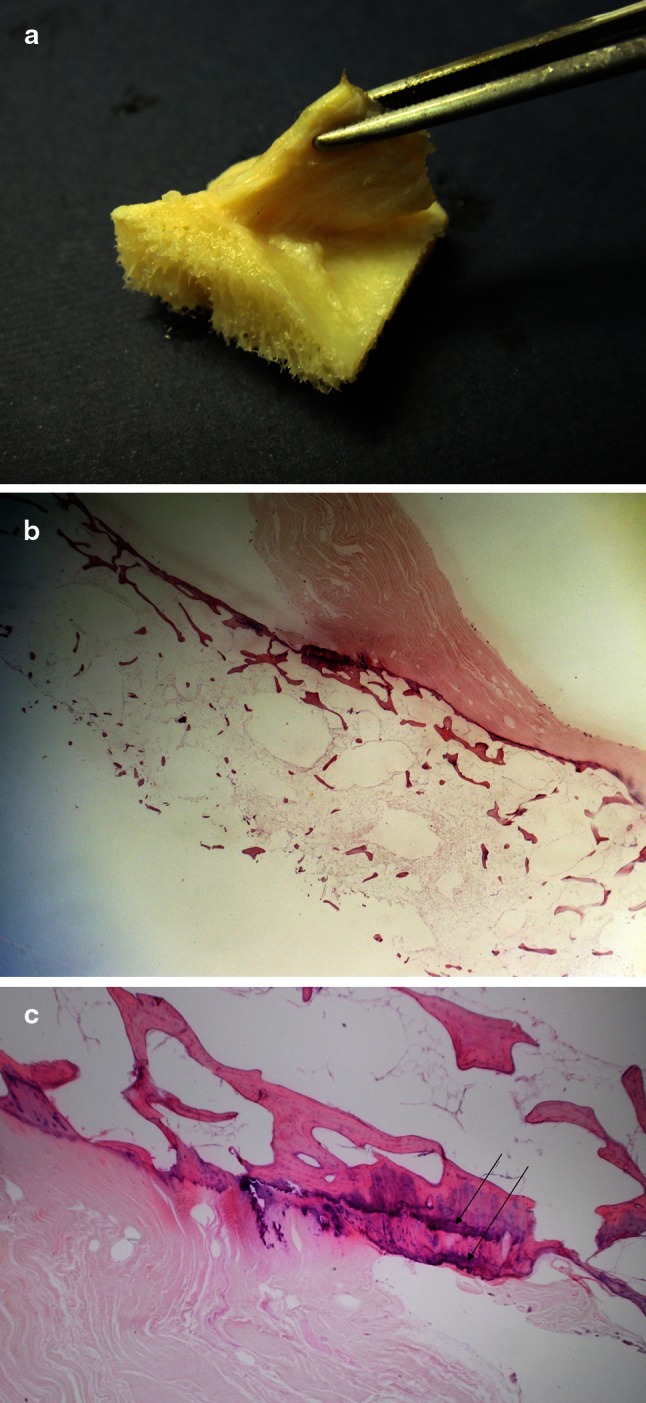
**a**–**b** Histology of the direct femoral insertion of the ACL: macroscopic view (**a**) and microscopic view (**b**, **c**), (light microscopy, H&E stain, original magnification ×4). **b** Note the sharp angle at which the fibres attach to the bone. Notice **c** double tidemark—marked with *arrows*

3D-CT reconstruction, MRI and histology reconfirmed the ribbon-like structure of the ACL. The collagen fibres were formed like a ribbon and approached to the femoral insertion in an acute angle creating a doubled tidemark at the bone (Fig. 4a, b).

This may be interpreted as a place within the whole attachment with either greater stress forces or microinjuries [7]. In both interpretations that would be the place where the greatest force is applied and therefore that would be the place where our graft should arise from ist tunnel.

## Discussion

The most important finding of this study was that the ACL formed a flat ribbon-like ligament from its femoral insertion to midsubstance in all dissected knees. The ACL fibres were in exact continuity with the posterior femoral cortex and inserted just posterior to the intercondylar ridge. A clear separation into bundles was not possible. Anatomical observations were based on dissections of 111 cadaveric knees and were reconfirmed on CT, MRI and histologically.

Earlier reports from the literature are reconfirmed by our findings. In 2006, Mochizuki et al. [31] emphasised “that—after removal of the surface membrane—the configuration of the intraligamentous part of the ACL was not oval” but rather flat, looking like “lasagna” 15.1 mm in length and 4.7 mm in width. Mochizuki et al. [31] also described the femoral insertion of the ACL to be very similar to the midsubstance configuration after the ligament surface membrane was removed from the attachment site. In 2010, Iwahashi et al. [25] reported on the “direct” femoral ACL insertion in which dense collagen fibres were connected to the bone by a fibrocartilaginous layer. This “direct” insertion was located in the depression between the lateral intercondylar ridge and 7–10 mm anterior to the articular cartilage margin. It measured 17.9 mm in length and 8.0 mm in width and covered an area of 128.3 mm^2^. These findings were reconfirmed by Sasaki et al. [42] who observed a narrow “direct” ACL insertion area posterior and along the lateral intercondylar ridge and a “lateral intercondylar posterior ridge”. The lengths of the long and short axes of the insertion were 17.7 and 5.3 mm, respectively. Another “indirect” ACL insertion was located just posterior to the direct insertion. The ACL from type I collagen blended into the posterior cartilage on immunohistological observations [42].

In a second report, Mochizuki et al. [30] just recently differentiated between the main attachment of the midsubstance ACL fibres and the attachment of the thin fibrous tissue. Later extended from the midsubstance fibres and broadly spread out like a fan on the posterior condyle. The authors termed these fibres ‘‘fan-like extension fibres’’ and described that these two different structures formed a fold at the border between the midsubstance fibres and the fan-like extension fibres in knee flexion.

MRI measurements were taken in 30 specimens, and reports from the literature also reconfirmed the flat ribbon-like midsubstance ACL. Staeubli et al. [47] measured the midsubstance in 53 knees using a 0.23 T MRI and found a width of 6.1 mm in men and 5.2 mm in women, Muneta et al. [34] reported 5.5 and 5.1 mm, respectively, and Pujol et al. [40] 6.1 mm. Cohen et al. [9] scanned the knees of 50 patients using a 1.5 T MRI and measured the dimensions of the AM and PL bundles in the sagittal and coronal plane to be 5.1 mm by 4.2 mm (AM) and 4.4 mm by 3.7 mm (PL).

The cross-sectional area of the midsubstance ACL was calculated and measured 52 and 55 mm^2^ for woman and men approximately 2 mm to its femoral insertion site and 33 and 38 mm^2^ at midsubstance, respectively. This is in agreement with several previous reports. Mochizuki et al. [31] approximated 65 mm^2^ as the femoral attachment area, Harner et al. [21] calculated approximately 40 mm^2^ at midsubstance, Hashemi et al. 46.8 mm^2^ [22] and Iriuchishima et al. 46.9 mm^2^ [24]. Differentiating between gender Anderson et al. [4] calculated a cross-sectional area of 44 mm^2^ for men and 36.1 mm^2^ for woman, Dienst et al. [12] of 56.8 mm^2^ for men and 40–50 % less for women on MRI and Pujol et al. [40] of 29.2 mm^2^ (range 20.0–38.9 mm^2^).

Bundles could not clearly be separated from our dissections. This is in agreement with Welsh [49] and Arnoczky [5] and others reporting that the intraligamentous part of the ACL is a collection of individual fascicles that fan out over a broad-flattened area with no histological evidence for two separate bundles [5, 11, 13, 26, 36, 49]. However, the recent approach to the ACL is to differentiate between anteromedial and posterolateral bundle [1, 6, 8, 14, 18–21, 29, 33, 45, 50] Some authors even described three separate ACL bundles [2, 35, 37]. The separation of the ACL into an AM and PL bundle was reconfirmed by Ferretti et al. [16], which found a fine synovial septum in dissected ACLs of foetus.

In any case, the macroscopic anatomical separation of the ACL into two or three bundles remains very difficult and is controversial. According to Arnoczky et al. [5], the bundle anatomy oversimplifies somewhat as the ACL is actually a continuum of fascicles. In 1991, Amis and Dawkins [2] described that it was sometimes difficult to separate the ACL into three discrete bundles. In these cases, the anterior aspect of the ACL was folded itself in flexion suggesting an arrangement of bundles. It was still possible to develop a three-bundle structure corresponding to the folding, but it felt that the teasing apart was artefactual. “In older specimens, however, the separate bundles were often obvious”. Amis and Dawkins [2] concluded that the ACL wrinkles into the appearance of three bundles as the knee flexes. These bundles are often demonstrably separate structures, twisted together during flexion, but the use of the dissector to separate the fibre bundles can cross the threshold between demonstration of bundles and their creation.

From our observation, the “double bundle effect” was created by the twisted flat ribbon-like structure of the ACL from femoral to tibial, which lead to the impression of two or three separate bundles when the knee was flexed. This would reconfirm reports of Amis and Dawkins [2] who made similar observations.

The ribbon-like shape of the ACL and the flat but long femoral “direct” insertion site would support a rather flat anatomical footprint and midsubstance reconstruction. A double bundle ACL reconstruction using two 5–6 mm hamstring grafts [25, 31, 32, 42, 44, 46], a flat 5–6 mm patella tendon graft [43] or a flat 5–6 mm quadriceps tendon graft may be a better anatomical option than a large (and too wide) diameter graft for a single-bundle ACL reconstruction. Sasaki et al. [42] concluded that whereas the indirect insertion plays a role as a dynamic anchorage of soft tissue to bone allowing certain shear movements, the strength of anchoring is weaker than the direct insertion [48]. Therefore, it would be ideal to make the femoral tunnel on the direct insertion in the native ACL [42]. Mochizuki et al. [30] found that it is very difficult to reconstruct the fan-like indirect extension fibres by a bone tunnel; however, the midsubstance fibres of the ACL can be reconstructed. Of course, the most efficient anatomical and biomechanical ACL reconstruction has still to be proven in prospectively designed clinical long-term studies.

Our study was limited by the fact that all dissections were performed by the same investigator. Dissections have been done without magnification under direct visualisation, and the morphometric measurements were performed directly at the ligament using a calliper. However, the results of our macroscopic dissections are supported by radiological, histological findings and recent findings from the literature.

The clinical relevance of this study might have an impact on surgical technique used for anatomical ACL reconstruction.

## Conclusion

This is a detailed anatomical study describing the ribbon-like structure of the ACL from its femoral insertion to midsubstance. A key point was to carefully remove the surface fibrous membrane of the ACL. Two millimetre from its bony direct femoral insertion the ACL formed a flat ribbon-like ligament without a clear separation between AM and PL bundles. The ribbon was in exact continuity of the posterior femoral cortex. The findings of a flat ligament may change the approach to femoral ACL footprint and midsubstance ACL reconstruction and to graft selection.

## Electronic supplementary material

Below is the link to the electronic supplementary material.

Supplementary material 1 (MPG 16578 kb)
